# Cosmopolitan Species As Models for Ecophysiological Responses to Global Change: The Common Reed *Phragmites australis*

**DOI:** 10.3389/fpls.2017.01833

**Published:** 2017-11-16

**Authors:** Franziska Eller, Hana Skálová, Joshua S. Caplan, Ganesh P. Bhattarai, Melissa K. Burger, James T. Cronin, Wen-Yong Guo, Xiao Guo, Eric L. G. Hazelton, Karin M. Kettenring, Carla Lambertini, Melissa K. McCormick, Laura A. Meyerson, Thomas J. Mozdzer, Petr Pyšek, Brian K. Sorrell, Dennis F. Whigham, Hans Brix

**Affiliations:** ^1^Aquatic Biology, Department of Bioscience, Aarhus University, Aarhus, Denmark; ^2^Institute of Botany, The Czech Academy of Sciences, Průhonice, Czechia; ^3^Department of Landscape Architecture and Horticulture, Temple University, Ambler, PA, United States; ^4^Department of Entomology, Kansas State University, Manhattan, KS, United States; ^5^Department of Natural Resources Science, University of Rhode Island, Kingston, RI, United States; ^6^Department of Biological Sciences, Louisiana State University, Baton Rouge, LA, United States; ^7^College of Landscape Architecture and Forestry, Qingdao Agricultural University, Qingdao, China; ^8^Institute of Ecology and Biodiversity, School of Life Sciences, Shandong University, Jinan, China; ^9^Department of Watershed Sciences and Ecology Center, Utah State University, Logan, UT, United States; ^10^Department of Agricultural Sciences, University of Bologna, Bologna, Italy; ^11^Smithsonian Environmental Research Center, Edgewater, MD, United States; ^12^Department of Biology, Bryn Mawr College, Bryn Mawr, PA, United States; ^13^Department of Ecology, Faculty of Science, Charles University, Prague, Czechia

**Keywords:** atmospheric CO_2_, climate change, eutrophication, global distribution, intraspecific variation, invasive species, salinity, temperature

## Abstract

*Phragmites australis* is a cosmopolitan grass and often the dominant species in the ecosystems it inhabits. Due to high intraspecific diversity and phenotypic plasticity, *P. australis* has an extensive ecological amplitude and a great capacity to acclimate to adverse environmental conditions; it can therefore offer valuable insights into plant responses to global change. Here we review the ecology and ecophysiology of prominent *P. australis* lineages and their responses to multiple forms of global change. Key findings of our review are that: (1) *P. australis* lineages are well-adapted to regions of their phylogeographic origin and therefore respond differently to changes in climatic conditions such as temperature or atmospheric CO_2_; (2) each lineage consists of populations that may occur in geographically different habitats and contain multiple genotypes; (3) the phenotypic plasticity of functional and fitness-related traits of a genotype determine the responses to global change factors; (4) genotypes with high plasticity to environmental drivers may acclimate or even vastly expand their ranges, genotypes of medium plasticity must acclimate or experience range-shifts, and those with low plasticity may face local extinction; (5) responses to ancillary types of global change, like shifting levels of soil salinity, flooding, and drought, are not consistent within lineages and depend on adaptation of individual genotypes. These patterns suggest that the diverse lineages of *P. australis* will undergo intense selective pressure in the face of global change such that the distributions and interactions of co-occurring lineages, as well as those of genotypes within-lineages, are very likely to be altered. We propose that the strong latitudinal clines within and between *P. australis* lineages can be a useful tool for predicting plant responses to climate change in general and present a conceptual framework for using *P. australis* lineages to predict plant responses to global change and its consequences.

## Introduction

One of the greatest challenges in ecology is to understand, predict, and mitigate the consequences of climate change (IPCC, 2014). Climate change will affect species interactions, community structure, and biodiversity, and will induce major shifts in plant phenology and geographic ranges (e.g., Post, 2013; Visser, 2016). However, not all species will respond similarly to changing climatic conditions (Springate and Kover, 2014). In a highly variable and changing environment, globally distributed species will likely have the genetic variation needed to acclimate to a broad spectrum of environmental and climatic gradients (Jump and Peñuelas, 2005). So far, however, most efforts to assess species changes have focused on climate modeling (e.g., Thuiller et al., 2005; Munguia-Rosas et al., 2011; Niu et al., 2014) or experiments using plants that are unlikely to have widespread impacts on community diversity or ecosystem processes (e.g., Chapman et al., 2014; Springate and Kover, 2014).

Species with the high genetic diversity and heritable phenotypic variation typically seen in cosmopolitan species are likely to have more inherent flexibility to evolve in response to climate change than species with low intraspecific diversity and restricted geographic ranges (Lavergne and Molofsky, 2007). Moreover, genotypes with high phenotypic plasticity (i.e., a high capacity of a genotype to produce distinct phenotypes in response to environmental variation; Bradshaw, 1965) typically have a greater capacity to adapt to altered environmental conditions than species with low plasticity (Franks et al., 2014; Valladares et al., 2014). Despite the fact that intraspecific variation is the basis of evolutionary change (Hiesey et al., 1942), it has only recently gained notice in studies of species responses to global change (Violle et al., 2012; Aspinwall et al., 2013; Pauls et al., 2013; Meyerson et al., 2016a; Münzbergová et al., 2017). Widespread and genetically diverse species, including those that are invasive, may be buffered against the adverse effects of global change (Oney et al., 2013). Truly cosmopolitan species, such as *Phragmites australis* (Cav.) Trin. ex Steud. (common reed), have global distributions, high genetic and phenotypic variation, and occur in a wide range of environments. The high intraspecific diversity usually found within *P. australis* stands may provide the species with the ability to cope with and benefit from a rapidly changing climate (Jump and Peñuelas, 2005; Kettenring et al., 2010, 2011). However, some populations may experience decreased genetic diversity during the acclimation and adaptation processes (Almeida et al., 2013). At the community and ecosystem scales, local extinction (Bolnick et al., 2011) and the alteration of small-scale environmental conditions and species-interactions (Crutsinger et al., 2008; Schöb et al., 2013) may be the ultimate consequences of the loss of intraspecific diversity. Whilst it is highly unlikely that species with high intraspecific diversity could be threatened with total extinction, shifts in genetic composition, including the genetic impoverishment of a population, may occur (Franks et al., 2014; Valladares et al., 2014). Therefore, a key challenge awaiting future research is determining how intraspecific variation drives local species composition and mediates the effects of rapid environmental change.

*Phragmites australis* is a cosmopolitan species that has strong effects on the ecosystems it inhabits; it therefore can offer valuable insights into plant responses to global change (Den Hartog et al., 1989; Chambers et al., 1999; Koppitz, 1999; Engloner, 2009; Mozdzer and Megonigal, 2012; Caplan et al., 2015; Hughes et al., 2016). It is a robust and highly productive grass in the Poaceae family that occurs in a wide range of freshwater and brackish wetlands (Brix, 1999a; Meyerson et al., 2000) spanning temperate and tropical regions (Den Hartog et al., 1989). The success of *P. australis* as a cosmopolitan species is related to its high productivity, its rapid stand-scale expansion through both clonal and sexual reproduction, and its ability to evolve rapidly in new ranges (Kettenring et al., 2010, 2011, 2012, 2015; Douhovnikoff and Hazelton, 2014; Eller et al., 2014a; Saltonstall et al., 2014). Changes in the distribution and growth patterns of *P. australis* have strong socioeconomic and environmental impacts that may be influenced by, and also feedback on, changing climatic conditions (Kim et al., 1998; Dukes and Mooney, 1999; Brix et al., 2001; Windham and Meyerson, 2003). The species has undergone an almost exponential range-expansion in North America (Chambers et al., 1999), where it is considered one of the worst invasive species on the continent (Saltonstall, 2002; Hazelton et al., 2014). Its global distribution and ability to proliferate in a wide range of habitats, especially in areas where physical disturbances are abundant, appear to derive from its distinct ecophysiological strategies, broad ecological amplitude, high evolutionary potential, and high phenotypic plasticity (Eller and Brix, 2012; Kettenring and Mock, 2012; Mozdzer and Megonigal, 2012; Mozdzer et al., 2013; Guo et al., 2014; Kettenring et al., 2015, 2016; Bhattarai et al., 2017a; Packer et al., 2017b). Like other cosmopolitan invasive plant species (Lavergne and Molofsky, 2004), *P. australis* has recently been suggested as a model organism for studying plant invasions (Meyerson et al., 2016b; Packer et al., 2017a). Given its highly plastic physiological and morphological responses to interacting global change factors (Eller and Brix, 2012; Mozdzer and Megonigal, 2012; Eller et al., 2013, 2014a,b; Caplan et al., 2015), *P. australis* may also provide insights into global change responses of other plant species.

Despite the large body of knowledge generated by prior research on *P. australis*, it is perhaps surprising that there is no global synthesis of the genetic variability of *P. australis*, its functional traits, its ecophysiology, and how the performance of the species is expected to change in a rapidly changing environment, especially under the expected scenarios of global climate change. Our goal here is to provide a comprehensive review of the high intraspecific variation of the ecophysiological processes that allow *P. australis*, as a cosmopolitan species, to respond to global change factors such as temperature, atmospheric CO_2_ concentrations, drought, flooding, salinity, and eutrophication. We further aim to highlight the value of *P. australis* as a model species both for plant invasions, a widespread phenomenon with accelerating dynamics (van Kleunen et al., 2015; Pyšek et al., 2017) and also for cosmopolitan species’ responses to environmental change. Moreover, our review identifies and resolves knowledge gaps to further elucidate plant responses to global change.

## Intraspecific Variation

**FIGURE 1:**
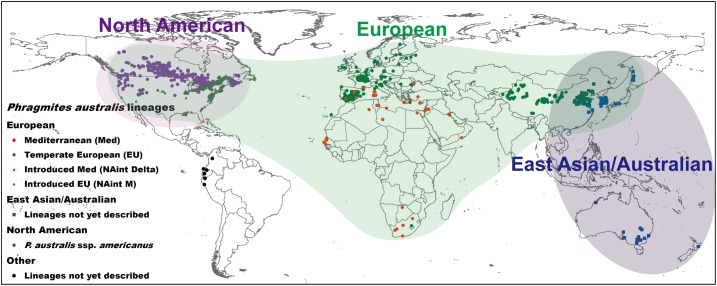
Global distribution of three main phylogeographic groups (North American, European, and East Asian/Australian) of the cosmopolitan wetland grass *Phragmites australis*, including several distinct lineages within the groups. More lineages or groups could possibly exist but have not been described yet. Points represent the collection locations of herbarium specimens analyzed by Lambertini et al. (2012c) and Guo et al. (2013) as well as the collection locations of several additional specimens at the Aarhus University herbarium.

Although *P. australis* is classified as one species, it is comprised of three main phylogeographic groups. These can be identified by their chloroplast DNA sequences (Lambertini et al., 2012c) and include: (i) the North American group, which contains *Phragmites australis* subsp. *americanus* (hereafter NAnat; Saltonstall, 2002), (ii) the East Asian/Australian group, and (iii) the Northern Hemisphere/African group (**Figure 1**). *Phragmites australis* of the latter region is known as European *Phragmites* (*sensu* Lambertini et al., 2012c) and is poised to benefit the most from global change. It has recently enlarged its geographic range via two invasive lineages. European *Phragmites* includes the lineages “EU” in temperate Europe and elsewhere, “Med” in the Mediterranean region of Europe and north and south Africa (Lambertini et al., 2012c; Guo et al., 2013), and their introduced lineages in North America. The introduced lineages are known as “Haplotype M” (hereafter NAint M), which occurs across the North American continent in sympatry with NAnat, and the “Delta-type” (NAint Delta), which occurs in the Mississippi River Delta and in isolated populations in Florida (Lambertini et al., 2012b). Populations of the invasive lineages are genetically and ecophysiologically distinct from their native populations in Europe (Saltonstall, 2002; Lambertini et al., 2012b,c; Tho et al., 2016). They are reported in the literature under these specific names, which is why they are referred to here as NAint M and NAint Delta. European *Phragmites* also occurs across the continents of Africa and Asia in sympatry with other *Phragmites* species and *P. australis* lineages of the East Asian/Australian phylogeographic group in East Asia. The ranges of the *P. australis* East Asian/Australian and North American groups have been more stable than the range of European *Phragmites*. However, this pattern might reflect isolation or a lower research effort rather than these genotypes having lower fitness to establish in new ranges. More lineages have been found outside of the three groups, but these are not well-described and consist of scattered observations, or are *Phragmites* species other than *P. australis* (**Figure 1**). In the absence of an updated revised systematics reflecting the genetic structure of the species, we use the above names to refer to the above described lineages and phylogeographic groups of *P. australis*.

*Phragmites australis* lineages and genotypes can be very diverse within and among populations, and genes from relatives in other phylogeographic regions or species can become incorporated into populations. This is due to a combination of inter- and intraspecific hybridization (McCormick et al., 2010a; Meyerson et al., 2010b; Chu et al., 2011; Paul et al., 2011; Lambertini et al., 2012b,c; Saltonstall et al., 2014; Saltonstall and Lambert, 2015; Wu et al., 2015), polyploidy (Clevering and Lissner, 1999; Meyerson et al., 2016a), genome size variability (Suda et al., 2015; Meyerson et al., 2016a), heteroplasmy (Lambertini, 2016), and long-distance dispersal.

## Influences of Environmental Gradients and Phenotypic Plasticity on *P. australis* Phenotypic Diversity

The phenotypic diversity of globally dispersed species derives from adaptations to environmental factors such as climate or day length; phenotypes are therefore expected to vary over broad latitudinal ranges (Wilson, 1988; Coomes and Grubb, 2000; Poorter et al., 2009). Differences among distinct lineages of *P. australis* reflect adaptations to the environment of their geographic origin and include differences in plant traits, the degree of phenotypic plasticity, and the environmental drivers to which these traits respond (Eller and Brix, 2012; Mozdzer and Megonigal, 2012; Eller et al., 2013; Mozdzer et al., 2013, 2016a,b; Bhattarai et al., 2017a).

Phenotypic differences within *P. australis* are apparent along clines within lineages and phylogeographic groups (Bastlová et al., 2006; Reich and Oleksyn, 2008; Cronin et al., 2015; Mozdzer et al., 2016a; Allen et al., 2017; Bhattarai et al., 2017a). A general observation is that shoots increase in height with decreasing latitude and altitude (Haslam, 1973; Clevering et al., 2001; Hansen et al., 2007; Mozdzer et al., 2016a), but these trends are non-linear across broad latitudinal ranges (Mozdzer et al., 2016a). In the Mediterranean region *P. australis* can reach heights of up to 5 m, while temperate European *Phragmites* usually has stem heights of 2–3.5 m (Haslam, 1972; Eid et al., 2010; Packer et al., 2017b). European *Phragmites* populations from lower latitudes allocate relatively little biomass to leaves and more to stems; they also produce fewer shoots than populations originating from higher latitudes (Hansen et al., 2007; Eller and Brix, 2012). Also, northern populations have an earlier onset of flowering, a shorter growing season, and greater resistance to winter frosts, which is even more pronounced in populations from continental climates (Clevering et al., 2001; Bastlová et al., 2006; Lambertini et al., 2012c). On the local scale, water availability and soil properties such as salinity are important controls of *P. australis* morphology and biomass; this derives from the high phenotypic plasticity of the species (Vretare et al., 2001; Achenbach et al., 2013; Hughes et al., 2016; Mozdzer et al., 2016a). Plastic and genetically determined differences in *P. australis* below-ground structures yield considerable differences in seasonal shoot initiation, root organic acid content, rhizome construction costs, and rhizospheric microbial communities (Dykyjová et al., 1970; Moore et al., 2012; Zhai, 2013; Caplan et al., 2014).

Several ploidy levels have been identified in *P. australis* genotypes, specifically 2n = 3×, 4×, 6×, 8×, 10×, 12× (Gorenflot et al., 1983). Higher ploidy levels often result in larger plants (Stebbins, 1971, but see Meyerson et al., 2016b). However, only the octoploids from Romania, belonging to European *Phragmites*, have been found to have giant traits compared to the other ploidy levels (Hansen et al., 2007; Achenbach et al., 2012). In the Danube Delta, the octoploids have bigger leaves, are taller, and have thicker shoots than the tetraploids (Rodewald-Rudescu, 1974; Hanganu et al., 1999; Pauca-Comanescu et al., 1999; Clevering et al., 2001). However, gas exchange rates are not affected by differences in ploidy level (Hansen et al., 2007; Saltonstall and Stevenson, 2007), and neither are salt tolerance or a range of growth and ecophysiological traits (Achenbach et al., 2012, 2013). This suggests that ploidy level has a minor or still poorly understood role in determining phenotypic characteristics within the species, particularly when it interacts with genome size (Meyerson et al., 2016a).

## Intraspecific Diversity Determines Responses to Global Change Drivers – The CRC (Cause-Response-Consequence)-Model

**FIGURE 2:**
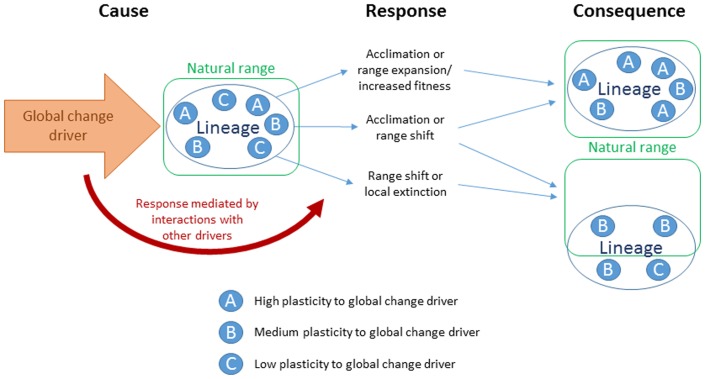
CRC (cause-response-consequence) model of global change driver acting upon lineages (or geographic populations within a lineage) composed of different genotypes. A global change driver affects the lineage which consists of highly plastic genotypes with respect to the driver **(A)**, moderate plasticity with respect to the driver **(B)**, and low plasticity with respect to the driver **(C)**. Plasticity refers to phenotypic plasticity in fitness-related traits (reproduction and productivity), thus affecting the genotype’s acclimation and adaptation capacity. The genotypes respond differently to the driver depending on their phenotypic plasticity; likely responses are acclimation, increased fitness, range expansion, range shift, or local extinction. Acclimation is the response to the environmental driver that results in similar or increased fitness. This scenario will likely lead to range expansion. A range shift occurs from the natural range of occurrence, which is the current distribution range including the native range for native lineages and the presently invaded range for introduced lineages. The responses can be mediated by interacting environmental drivers. The ultimate consequence of the responses to the effect are impoverished genetic diversity, including lineages with lower phenotypic plasticity and fewer, but better adapted genotypes, or a lineage shifting into a new range less or differently affected by the global change driver.

**FIGURE 3:**
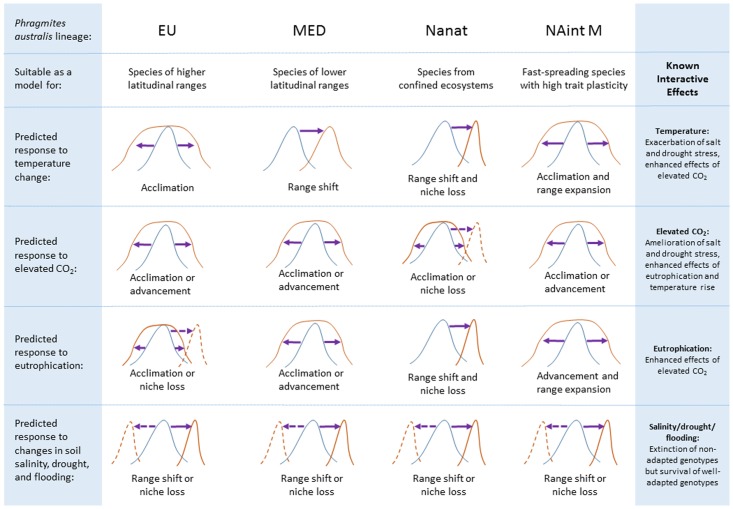
Specific effects of global change drivers on reed lineages. Lineage response is averaged, based on studies conducted on several genotypes from within these lineages. Well-established interactions with other global change drivers are specified. Curves show ecophysiological amplitude with specific niche-breadth and response strength to changes. Each lineage response can be extrapolated to different species with similar ecophysiological characteristics. Curves outline a relative normal distribution of fitness-related parameters of the population. A narrower curve means a narrower niche-breadth with respect to a global change factor (on *x*-axis). Advancement here means increased fitness. Blue curves show the current stage while orange curves result from the action of the specific global change factors. Either solid or dashed curve are expected to appear, but not both simultaneously.

Dominant and invasive species can modify community traits and ecosystem processes (e.g., species richness or primary productivity), thereby affecting regional and biogeographic patterns of species distribution and interactions (Wright and Jones, 2004; Vilà et al., 2011; Pyšek et al., 2012; Hughes et al., 2016). High genetic diversity provides *P. australis* with a broad ecological amplitude, which may be especially important when it colonizes new habitat or faces environmental stresses (van der Putten, 1997; Clevering, 1999; Koppitz, 1999). The capacity of *P. australis* to acclimate and eventually adapt to environmental change depends not only on the degree and nature of the change, but also on the genetic composition of the lineage itself (Hiesey et al., 1942; Eller and Brix, 2012). A lineage can be described as an entity consisting of several genetically distinct genotypes, each of which shares a part of the genome with the genotypes of the same lineage, but is also comprised of different genes and phenotypic plasticity toward various environmental drivers (Bradshaw, 1965). Phenotypic plasticity is a genetically determined trait-set, and recent studies have shown that plastic responses are inheritable and determined by the climatic origin of a plant (Latzel and Klimesova, 2010; Münzbergová and Hadincová, 2017). The sum of all plastic responses of a geographic population within a lineage is determined by genotypes responding to a specific environmental factor (**Figure 2**). Genotypes with high or medium plasticity toward a specific driver of environmental change will be able to acclimate to that driver, meaning that they will thrive equally well before vs. after the change. Hence, a population consisting of mainly highly or moderately plastic genotypes will change in genetic composition and the resulting population will consist of genotypes able to thrive under the changed conditions. Genotypes with low plasticity toward that specific driver will be subject to local extinction or a range shift if a more suitable habitat without the change is accessible for establishment (**Figure 2**). Global drivers of spatially homogeneous impact, such as the concentration of atmospheric CO_2_, therefore pose a greater challenge than patchy changes such as soil salinity. Some *P. australis* lineages show predictable responses to climatic and environmental scenarios, and are therefore particularly suitable models for understanding and predicting adaptation processes and evolutionary dynamics in other plants and plant types. We describe below the ecophysiological responses to global change drivers and present a conceptual model (**Figure 3**) that predicts how each *Phragmites* lineage will evolve by acclimation and adaptation to the drivers. Some reed lineages have not been described well enough in the literature to be included in the model, such as NAint Delta and the Far East/Australian (FEAU) group. The FEAU group is likely to be a suitable model for highly productive species like tropical grasses, but needs further investigation, especially with respect to phenotypic plasticity.

The conceptual model presented in **Figure 3** is based on the responses of *P. australis* lineages to factors associated with global change that act upon a lineage individually or in combination (**Table 1**). Overall, EU and NAint M are the lineages best adapted to withstand temperature changes and, together with the MED lineage, elevated CO_2_, while MED and NAint M will respond most positively to eutrophication (**Figure 3**). NAnat is the lineage with the least acclimation capacity. However, interactions with other environmental factors may change the above predictions (**Figure 3**). In the following sections, we review the main ecophysiological processes of *P. australis* to illustrate the diversity of these processes as a function of intraspecific variation and phenotypic plasticity, as well as the breadth of ecological niches that the species inhabits. We further describe ecophysiological responses to environmental factors to which the species is commonly exposed: temperature, atmospheric CO_2_ concentration, salinity, flooding, drought, and eutrophication. All of these factors are currently changing and are expected to change further in upcoming decades (IPCC, 2014). We also show how and why *P. australis’* responses to global change can be extrapolated to predict those of other species.

**Table 1:** Group or lineage specific responses of *Phragmites australis* to global change factors.

	European *Phragmites*		North American *Phragmites*			Asian/Australian *Phragmites*
			
Lineage	EU	Med	NAint M	NAint Delta	NAnat	Not defined
*Natural temperature range*	Average monthly temperature for survival -14 to 27.5°C (Haslam, 1975; Clevering et al., 2001; Gorai et al., 2006); shoot emergence and germination from -2 to 8°C (Haslam, 1975; Irmak et al., 2013); optimum temperature: 20 to 30°C (Haslam, 1975; Gorai et al., 2006)		Annual mean temp on average 7°C (Guo et al., 2013)	Annual mean temp on average 18 to 20°C (Guo et al., 2013)	Annual mean temperature on average 4°C, ranging from 25 to -17°C (CliMond dataset in Kriticos et al., 2012)	18 to 32°C mean annual warmest temp, 0 to 15°C mean annual coldest temperature in Japanese vs. Australian populations (Karunaratne et al., 2003)
	Annual mean temperature on average 10°C (Guo et al., 2013)	Annual mean temperature 18 to 20°C (Guo et al., 2013)				

Elevated temperature	Germination suppressed above 30°C (Haslam, 1975; Gorai et al., 2006); temperature fluctuation results in stimulated shoot growth and germination (Haslam, 1975; Brix, 1999a); lower photosynthetic capacity and Rubisco activity but increased growth (Eller et al., 2013)		Strong growth- and photosynthetic response to elevated temperature, if growth-CO_2_ concentration is elevated concomitantly (Eller et al., 2014a)		*No investigations found*	>25°C decline of photosynthetic parameters (Ge et al., 2014)
	Increased photosynthetic rates (Lessmann et al., 2001), high phenotypic plasticity to temperature (Eller and Brix, 2012)	Lower phenotypic plasticity to temperature compared with EU lineage (Eller and Brix, 2012)	Increased distribution toward higher latitudes due to seedling survival in warmer winters (Brisson et al., 2008)	Adapted and expanding to regions of high annual mean temperature (Guo et al., 2013)		

Elevated CO_2_	No effect on aboveground biomass, shoot or leaf production rates and shoot length, but increased photosynthetic capacity and Rubisco activity (Eller et al., 2013), lowered isoprene emissions (Scholefield et al., 2004)		Strong growth- and photosynthetic response to elevated growth-CO_2_ concentration if temperature is elevated concomitantly (Eller et al., 2014a)		Mildly increased biomass production (Mozdzer and Megonigal, 2012)	*No investigations found*
			Strong (37%) stimulation in *A*_sat_ with elevated CO_2_, which increased to 56% with CO_2_ + N (Mozdzer and Caplan, unpublished data). Effects of CO_2_ are driven by changes in physiology and morphology (Mozdzer and Caplan unpublished data)			
			Increased deep root production (Mozdzer et al., 2016b), strongly increased biomass production, especially after concomitant N addition (Mozdzer and Megonigal, 2012), amplified productivity throughout the growing season (Caplan et al., 2015)			

*Natural salinity range*	0 to 18 ppt, local adaptation of populations (Engels and Jensen, 2010; Achenbach et al., 2013)	0.3 to 27 ppt, fresh water, brackish water, mesophytic, sand dune and salt marsh habitats (Nada et al., 2015)	3.6 to 6.7 ppt (Yarwood et al., 2016), up to 30 ppt, survival from 7 to 24 ppt (Burdick and Konisky, 2003; Vasquez et al., 2005)	*No reports found*	2.6 to 6.2 ppt (Yarwood et al., 2016), survival from 1.2 to max. 18 ppt (Vasquez et al., 2005), no differences in growth performance from 03 to 12 ppt (Price et al., 2014), mesohaline wetlands (Meyerson et al., 2010a,b)	Growth at 0.9 to 28 ppt (Gao et al., 2012; Ma et al., 2013), seed germination <30 ppt but highest <20 ppt (Yu et al., 2012), local adaptation of reeds occurring from <6 ppt to >18 ppt (Holmes et al., 2016); 6 to 7 ppt healthy adult stands (Li et al., 2013)

Increased salinity	If originating from freshwater marsh, reed will have declined biomass and survival in salt marshes (Engels and Jensen, 2010)	Stable water-use efficiency and only slightly lower photosynthetic rates, also depending on nutrient and water availability in natural habitat (Nada et al., 2015)	Lower expression of photosynthetic genes, somewhat increased expression of stress-related genes (20 ppt; Eller et al., 2014b)		Considerably lowered growth and survival, more than NAint M (Vasquez et al., 2005)	Seed germination decreases above 30 ppt (Yu et al., 2012), slightly (15 ppt) and severely (30 ppt) decreased photosynthetic rates (Ge et al., 2014)
			Better performance with higher salinity in natural marshes (Vasquez et al., 2005; Price et al., 2014), increased expansion into oligo- and mesohaline marshes (Chambers et al., 1999)	High salt tolerance in laboratory (20 ppt), especially when temperature and CO_2_ are elevated (Eller et al., 2014a)		
			Salinity increased stimulation effects of elevated CO_2_ in the field up to 18 PSU (Mozdzer and Caplan, unpublished data)			

Drought	In fluctuating water-levels and short-term drought events, whole-plant leaf-area decreases to maintain high assimilation rates in the remaining leaves (Saltmarsh et al., 2006)		Lower seed production and height growth (Minchinton, 2002; Price et al., 2014)	*No reports found*	Although inland ecotypes predominate in the arid regions of the Southwest, groundwater drawdown is a threat (Meyerson et al., 2010a,b)	Ecotypes adapted to habitats of different water availability and also heavy drought stress, through gene expression, photosynthetic adaptations, and changed redox status (Wang et al., 1998; Chen et al., 2003; Gong et al., 2011; Zhu et al., 2012)
	High intrinsic water-use efficiency, leaf shedding and physiological maintenance of surviving leaves as tolerance method (Pagter et al., 2005)	Accumulation of compatible solutes increases from flooded to drained physiological maintenance of surviving leaves as tolerance habitats, little reduction in relative water content of leaves (Elhaak et al., 1993)				

Eutrophication	Weak culms susceptible to mechanical damage (most likely only EU lineage), suffering from anoxia in highly eutrophicated habitats (Cizkova-Koncalova et al., 1992; Meriste et al., 2012), but also increased growth (Kolada, 2016) or at least no negative effects (Vermaat et al., 2016)		Higher biomass and leaf area than EU and MED under unlimited nutrient supply (Tho et al., 2016)		Good competitor under low nutrient availability, but poor under eutrophicated conditions in nature (Holdredge et al., 2010; Mozdzer and Zieman, 2010), weak response to nutrient increase (Saltonstall and Stevenson, 2007), but high nutrient removal efficiency (especially P) in constructed wetland (Rodriguez and Brisson, 2016)	Large biomass development (Karunaratne et al., 2003; Han and Cui, 2016)
			N extends phenology leading to greater C gain (Caplan et al., 2015)			
			N induces changes in morphology (leaf area, height, and leaf width) that contribute to performance moreso than physiological adaptation (Mozdzer and Caplan, unpublished data)			
	Lower phenotypic plasticity to nutrient availability than MED (Eller and Brix, 2012)	High phenotypic plasticity to nutrient availability (Eller and Brix, 2012)	High photosynthetic rates and increased rhizome productivity under high nutrient availability (Holdregde et al., 2010; Mozdzer and Zieman, 2010), increased aboveground growth and shoot production (Saltonstall and Stevenson, 2007), increased establishment, growth and seedling production (Sciance et al., 2016)			

Flooding	Permanent water-logging is detrimental (Saltmarsh et al., 2006; Gigante et al., 2014), relatively fewer flood-tolerant genoypes grow in deep water compared to the edge (Engloner and Szego, 2016)		Seedling establishment mainly in less-frequently flooded habitats (Kettenring et al., 2015)	*No specific studies found*	*No specific records found*	Flooding can both facilitate and hinder the growth and expansion of reed ecotypes (Lee and An, 2015; Wang et al., 2015)
	Juvenile stems have low flooding tolerance, rhizomes and shoots have to be undamaged to survive short-term flooding, flooding events determine reed dynamics in lakes (Ostendorp and Dienst, 2012)					


*EU and MED lineage are not always separated especially in early publications and can be considered “native European *Phragmites*.” The natural range of an abiotic factor shows the current range of distribution, which is the native range for EU, Med, NAnat and Asian/Australian, and the introduced range for the invasive North American *Phragmites*, NAint M and NAint Delta.*

## Key Ecophysiological Processes

### Gas Exchange

Like biomass production and morphology, gas exchange-related traits in *P. australis* are highly plastic. Within a phylogeographic region, the prevailing climatic conditions have the strongest effects on gas exchange rates (Lessmann et al., 2001; Hansen et al., 2007; Mozdzer et al., 2016a). Although the climate of the area of origin strongly affects physiological responses, there are also phylogeographic differences in potential responses to environmental change. For example, the NAint Delta lineage was less plastic in its ability to modify gas exchange parameters compared to the highly plastic NAint M lineage when grown across 14° of latitude (Mozdzer et al., 2016a). Furthermore, tropical and subtropical populations of *P. australis* have a higher photosynthetic capacity and photosynthetic pigment concentration than populations in the temperate zone (Nguyen et al., 2013). Similarly, NAint *P. australis* has a higher photosynthetic capacity and pigment concentrations than NAnat (Mozdzer and Zieman, 2010; Guo et al., 2014). Nguyen et al. (2013) proposed the existence of a diversified C_3_ pathway within *P. australis* that is modified to maintain high enzymatic efficiencies in tropical and Mediterranean climates but can be down-regulated to accommodate the lower temperature and irradiance of temperate regions.

Despite the typical C_3_-photosynthetic features displayed by *P. australis*, C_4_-like strategies have also been observed. A prominent sheath layer that is especially pronounced in young *P. australis* leaves surrounds the vascular bundles in the mesophyll, resembling the foliar Kranz anatomy of C_4_ plants (Henriques and Webb, 1989). However, due to the lack of chloroplasts in this layer, there is no functional correlation with true C_4_ plants (Henriques and Webb, 1989). Doubts about the photosynthetic pathway of *P. australis* have also emerged due to relatively high PEPcase activities, higher activities of the decarboxylating NADP-dependent malic enzyme (NADP-ME), and a possible C_3_–C_4_ intermediate pathway associated with ecotypes from arid or salt-affected habitats (Rintamaki and Aro, 1985; Zheng et al., 2000; Zhu et al., 2012). Most of the known C_4_ species occur in the Poaceae, in which C_4_-evolution has occurred independently several times and, thus, genes are present in *P. australis* that can rapidly develop C_4_ functions including the gene coding for NADP-ME (Christin et al., 2009).

Nevertheless, *P. australis* has, in most studies, been shown to possess characteristics typical of C_3_ plants, including a high Rubisco/PEPcarboxylase ratio, high photorespiration rates, and a high CO_2_ compensation point (Antonielli et al., 2002; Hansen et al., 2007; Eller and Brix, 2012). The photosynthetic pathway of *P. australis* therefore remains unresolved, as the range of the abovementioned studies suggests that the photosynthetic pathway may vary within the species. The distinct bundle sheath cells in *P. australis* leaves also raise the possibility of C_2_ photosynthesis, which is the evolutionary bridge between C_3_ and C_4_ photosynthesis (Sage, 2016); however, evidence of this possibility has yet to be found.

### Nutrient Acquisition

By far the greatest number of scientific studies on *P. australis* have been concerned with the species’ tremendous potential for nutrient removal, which makes it an ideal candidate species for wastewater treatment in constructed wetlands (e.g., Brix and Schierup, 1989; Brix, 1997; Bragato et al., 2006; Vymazal, 2013; Hernández-Crespo et al., 2016). Genetically determined differences in nutrient uptake and assimilation capacity result in distinct reed ecotypes with differences in productivity (Tho et al., 2016). Some ecotypes sustain high nutrient assimilation rates and high allocation to aboveground biomass, while others have high nutrient translocation rates to rhizomes for storage and thus high belowground biomass allocation (Kühl et al., 1997; Tripathee and Schäfer, 2014). Reed genotypes with dissimilar nutrient demands and productivity can thus grow at similar nutrient levels in naturally adjacent stands. Such distinct ecophysiological strategies confer greater population plasticity and performance to a genetically diverse stand compared to a monoclonal stand (Rolletschek et al., 1999). Pronounced differences in the nitrate uptake kinetics of distinct reed genotypes are possibly caused by distinct transcript abundances of nitrate transporter genes, and a likely reason for the genotypic differences in nutrient acquisition strategies (Araki et al., 2005). In general, *P. australis* is well-adapted for growth in nutrient-rich habitats (Mozdzer et al., 2010; Caplan et al., 2015) but can also acclimate to low nutrient availability by increasing the affinity for ammonium uptake (Romero et al., 1999; Tylova-Munzarova et al., 2005; Mozdzer and Megonigal, 2012).

### Gas Transport and Ventilation

Like almost all plants that can grow vigorously in habitats where soil saturation and flooding are common (Vartapetian and Jackson, 1997), *P. australis* aerates flooded tissues by transporting oxygen through a well-developed network of internal airspaces, or aerenchyma (Armstrong and Armstrong, 1991; Jackson and Armstrong, 1999). These internal airspaces are continuous from the leaf sheaths and culms, through the rhizomes, and into the root cortex, where aerenchyma are particularly well-developed through lysigeny (Armstrong et al., 1996a; White and Ganf, 2002). Rhizomes are segmented internally and have secondary aeration channels in the internode cortex, such that airflow is maintained even if rhizome cavities become damaged and filled with water (Soukup et al., 2000). More efficient root aeration also allows for greater respiration rates and, thus, sustained nutrient uptake capacity and root development, even in hypoxic soils (Nakamura et al., 2013).

*Phragmites australis* is also one of the few wetland species that does not rely solely on simple diffusion for gas transport; it supplements its aeration with convective gas flow (Brix, 1989; Brix et al., 1992, 1996; Armstrong et al., 1996a). Convection is induced by humidity gradients generated in lacunae (i.e., sub-stomatal cavities) in leaf sheaths of live culms (Armstrong et al., 1996a,c). The pressure that builds up in lacunae pushes air down through live culms and rhizomes; air is vented out of the plant through damaged or dead culms (Brix, 1989; Armstrong et al., 1996c; Afreen et al., 2007).

Little attention has been paid to potential intraspecific differences in gas transport among *P. australis* lineages. Tulbure et al. (2012) showed that the ventilation efficiency of the invasive NAint M lineage in North America was 300 times higher than that of native *P. australis* subsp. *americanus*, when differences in stem densities between lineages were accounted for. Since gas flux is a physically determined process and is strongly affected by internal anatomy (Rolletschek et al., 1999), different gas flow behavior can be expected in plants with genotype-specific morphological characteristics. Moreover, gas flow characteristics of wetland plants affect not only oxygen transport but also plant-mediated methane emission (Brix et al., 2001), and lineage-specific differences in factors controlling gas flow are known to affect methane fluxes (Armstrong et al., 1996b; Kim et al., 1998). For example, NAint M roots more deeply than other lineages and, through changes in soil organic matter dynamics, can lead to increased rates of CO_2_ losses to the atmosphere (Bernal et al., 2017). Differences in gas flow capacity and rhizosphere oxygenation among lineages are therefore very likely and deserve greater attention.

## Effects of Major Drivers of Global Change on the Performance of *P. australis*

Contrasting responses to global change drivers have been reported in North American and European *Phragmites. Phragmites australis* of Asia and Australia has received limited attention, so their responses to such drivers remain poorly understood. From the 1970s to the 1990s, *P. australis* in Europe experienced a decrease in abundance termed ‘reed dieback,’ largely due to anthropogenic eutrophication and deeper flooding, especially in Eastern Europe (Ostendorp, 1989; van der Putten, 1997; Brix, 1999b). Increased salinity caused by land use changes may also have contributed to reed dieback in northern European brackish marshes, as it may have allowed halophytes like *Spartina alterniflora* to displace less salt-tolerant species like *P. australis* (Vasquez et al., 2006). Reductions in *P. australis* growth have also been associated with litter accumulation leading to the production of phytotoxins (Armstrong et al., 1996a; Čížková et al., 1999) and high rates of anaerobic mineralization stemming from excess organic matter and the associated increase in biological oxygen demand (Sorrell et al., 1997). Degraded reed stands have been shown to have an altered C/N metabolism due to higher rates of photorespiration and thus, lower carbon fixation (Erdei et al., 2001).

In contrast to the situation in Europe, the species has shown invasive behavior in North America over the last 50 years. The invasion is driven by a few lineages originating from European *Phragmites* (Hauber et al., 1991; Saltonstall, 2002; Hauber et al., 2011; Lambertini et al., 2012b) and may depend largely on the high genetic diversity of the species in its native range (Saltonstall, 2003; McCormick et al., 2010b; Pyšek et al., 2017).

### Temperature Effects

Without considerable greenhouse gas reductions, the global rise in mean surface temperature of Earth is very likely to exceed 1.5–4°C by the end of the 21st century, with the greatest increases in the Northern Hemisphere (IPCC, 2014). Heatwaves and extreme precipitation events are expected to occur more frequently and with longer durations in many regions, but occasional cold temperature extremes can also be expected (IPCC, 2014).

*Phragmites australis* exhibits lineage-specific responses to temperature regimes in terms of morphology, growth, and to a certain extent, photosynthetic traits (**Table 1**; Clevering et al., 2001; Lessmann et al., 2001; Eller and Brix, 2012; Eller et al., 2013; Mozdzer et al., 2016a). Rates of *P. australis* growth (especially shoot height and length), as well as rates of transpiration and photosynthesis, are generally greater at lower latitudes due to warmer temperature regimes and longer day lengths (Haslam, 1975; Lissner et al., 1999a,b; Zemlin et al., 2000; Lessmann et al., 2001; Karunaratne et al., 2003; Mozdzer et al., 2016a). Reciprocal transplant experiments in common gardens have shown that, for lineages originating from lower latitudes, higher temperatures are needed to initiate growth and, after being transplanted to higher latitudes, panicles either emerge late or do not flower at all (Brix, 1999b; Clevering et al., 2001; Karunaratne et al., 2003; Lambertini et al., 2012c). Adaptation to the climate in the region of origin significantly affects plant species’ performance and plasticity (Franks et al., 2014; Molina-Montenegro et al., 2016; Allen et al., 2017; Bhattarai et al., 2017a,b; Münzbergová et al., 2017). Hence, *P. australis* belonging to the MED lineage can be a model for Mediterranean, subtropical, and even tropical plant species, while populations of the EU lineage can be a model for temperate species found at higher latitudes (**Figure 3**).

Some lineages seem to be more plastic to changes in temperature than others, as they show a large acclimation capacity to both increases and decreases in temperature (**Figure 3**; Lessmann et al., 2001; Eller and Brix, 2012). This is the case for NAint M in North America, for example, where temperature fluctuations have been shown to enhance its distribution (Guo et al., 2013). The North American invasion is therefore likely to accelerate with climate change. It has previously been suggested that lineages originating in areas with high fluctuating temperatures also have higher plasticity to temperature changes, and may therefore be better adapted to withstand climatic changes (Molina-Montenegro and Naya, 2012). The same has been shown for *P. australis* lineages; EU genotypes from higher latitudes in temperate areas have generally shown higher plasticity toward differences in growth temperature (Lessmann et al., 2001; Eller and Brix, 2012; Nguyen et al., 2013). It can be assumed that the high plasticity of NAint M derives from its origin in the highly plastic EU populations, emphasizing the potential model role of *P. australis* lineages from high-latitudes for temperate plant responses to temperature differences (**Figure 3**).

Other lineages appear to be pre-adapted to predicted future temperature regimes and are therefore likely to extend their range northward (Clevering et al., 2001; Lessmann et al., 2001; Eller et al., 2014a,b; Mozdzer et al., 2016a). It is possible that lineages originating from lower latitudes may expand their distributions northward in the warming world (**Figures 2**, **3**; Guo et al., 2013; Mozdzer et al., 2016a), as frost and cool temperatures limit growth or sexual reproduction at mid and high-latitudes (Mozdzer et al., 2016a). Also, the expansion of the invasive NAint Delta lineage can be attributed, in part, to warmer temperatures in its invasive range than in its native range (Guo et al., 2013), as advancement of a population can be expected if a high phenotypic plasticity to temperatures is inherent (**Figure 2**). Alternatively, lower-latitudinal lineages may be unable to cope with the rapidity of temperature changes due to a narrow niche-breadth or acclimation-capacity, and may become genetically diminished (**Figure 2**). Using *P. australis* as model for global warming, a two-way scenario can be expected as the species responds to temperature increases. On the one hand, species with high phenotypic plasticity, and therefore greater niche breadths, will likely be able to cope with warming and thrive equally well or even extend their range northward. Another species in which this is likey to occur is *Nothofagus pumilio* (Mathiasen and Premoli, 2016). On the other hand, species with limited plasticity and narrower niche breadths may fail to acclimate, facing local extinction in the worst case (**Figure 2**). Some herbaceous alpine species occurring at high elevation provide a good example of narrow niche breadth leading to local extinction (Schmid et al., 2017). Indirect changes and interactions of temperature with other abiotic factors, such as increased drought and salinity, may impose additional challenges on *P. australis* populations in already warm areas, possibly resulting in more favorable growth conditions at higher latitudes (**Figure 3**; Brix, 1999b; Eller et al., 2014a).

### CO_2_ Effects

Atmospheric CO_2_-equivalents are likely to exceed 720 ppm, and possibly reach 1000 ppm, by the late 21st century if greenhouse gas emissions are not restricted substantially (IPCC, 2014). As a C_3_ plant, *P. australis* will benefit from rising atmospheric CO_2_ concentrations, but there is growing evidence that the magnitude of its response may be lineage-specific due to differences in phenotypic and physiological plasticity (**Figure 3**). For example, in an experiment in which CO_2_ was elevated to ∼700 ppm, both the NAint M and NAnat lineage responded positively to CO_2_ elevation but the NAint M lineage had considerably greater plasticity in nearly every trait measured (Mozdzer and Megonigal, 2012; Caplan et al., 2014). In contrast, several studies focusing on other lineages of *P. australis* have found no significant effects of elevated CO_2_ on biomass or morphological parameters, though some photosynthetic enhancements have been reported (Scholefield et al., 2004; Milla et al., 2006; Kim and Kang, 2008; Eller et al., 2013). We note that these studies either did not measure below-ground biomass productivity or did not account for respiration rates, which may partly explain the lack of biomass stimulation by elevated CO_2_.

Based on the above, differential responses to elevated CO_2_ may result in lineage-specific shifts, increases in competitiveness and distribution changes. However, interactions with other abiotic factors such as salinity and nutrients make it more difficult to predict the effects of elevated CO_2_ in natural environments. For example, whilst shoot elongation rates are enhanced by elevated CO_2_ and temperature to similar degrees in both the invasive NAint Delta and the invasive NAint M lineages, the NAint Delta lineage outperforms the NAint M lineage when grown at 20‰ soil salinity (Eller et al., 2014a). The stronger growth response of NAint Delta is facilitated, in large part, by intrinsically greater photosynthetic rates (**Table 1**; Nguyen et al., 2013; Eller et al., 2014a). Overall, the strongest effects on growth and carbon assimilation rates are expected to result from changes in CO_2_ that are accompanied by increases in temperature or nutrient enrichment, especially nitrogen (N) (**Figures 2**, **3**; Mozdzer and Megonigal, 2012; Eller et al., 2013, 2014a,b; Caplan et al., 2015), which has been shown for other C_3_ species (Ainsworth and Rogers, 2007).

Changes induced by elevated atmospheric CO_2_ concentrations may influence ecosystem services in *P. australis* dominated wetlands. For example, elevated CO_2_ increases the methane emission rate of both NAnat and NAint lineages (Mozdzer and Megonigal, 2013), which may offset the net carbon fixation of *P. australis* wetlands that would otherwise be greenhouse gas sinks (Brix et al., 2001). Moreover, elevated CO_2_ induces greater belowground productivity and rooting depths in the NAint M lineage (Mozdzer et al., 2016b), which are likely to increase rates of belowground biomass accumulation and surface elevation gain. Such effects could enhance the ability of *P. australis* dominated wetlands to keep pace with sea level rise (Rooth et al., 2003; Caplan et al., 2015; Mozdzer et al., 2016b). Responses to elevated CO_2_ have predominantly been investigated through short-term studies and have only investigated a few *P. australis* lineages (including NAnat, NAint M, NAint Delta, EU, and Med; Eller et al., 2013; Mozdzer and Megonigal, 2013; Eller et al., 2014a,b); more research is needed to determine if enhancement of growth and methane emission rates apply to the whole species. Due to its high plasticity to atmospheric CO_2_ concentration, the NAint M lineage can be used as a model for studying the responses of invasive C_3_ species to elevated CO_2_ (**Figure 3**), as high phenotypic plasticity is a common trait in invasive species (Drenovsky et al., 2012; Gioria and Osborne, 2014; Colautti et al., 2017).

### Salinity Effects

Saltwater intrusion due to global sea level rise is becoming a major issue in both brackish saltmarshes and tidal freshwater wetlands (Beckett et al., 2016). Moreover, regions with high salinity and high evaporation rates are likely to become more saline, while regions of low salinity and high precipitation will become fresher, inducing greater extremes in salinity in wetlands globally (IPCC, 2014). Finally, some climate change models predict an increase in the intensity and frequency of tropical storms and hurricanes (e.g., Bender et al., 2010; Knutson et al., 2010), which may lead to flooding and salt intrusion in near-coastal habitats. Hence, soil salinity regimes are shifting such that salinity tolerance is of increasing importance to biotic communities in coastal ecosystems.

The ecological amplitude of *P. australis* extends from freshwater to saline tidal wetlands, with plants persisting at salinities as high as 65‰ (recorded in Delaware, eastern United States; Engloner, 2009), with tolerances of 22.5‰ (Lissner and Schierup, 1997) to 35‰ salinity reported for juveniles (Engloner, 2009) and a limit of 30‰ reported for seed germination (Yu et al., 2012). The mechanisms of salt tolerance in *P. australis* include Na^+^ exclusion or vacuolar compartmentalization, tissue dehydration or compatible osmotic solute accumulation, and increased gene expression of oxidative stress response enzymes (Matoh et al., 1988; Lissner and Schierup, 1997; Lissner et al., 1999a; Pagter et al., 2005; Vasquez et al., 2005; Achenbach and Brix, 2013; Achenbach et al., 2013; Eller et al., 2014b). Certain ecotypes of *P. australis* are more salt tolerant than others (**Table 1**), with higher salinities yielding greater germination rates and better developed root systems (Rechav, 1967; Van der Toorn, 1972). Several controlled experimental studies have shown that NAint Delta has higher salt tolerance than NAint M, though both perform better at higher salinities than Med or the NAnat lineages (Achenbach and Brix, 2013; Eller et al., 2014a). Since the NAnat lineage has considerably greater N uptake rates than the invasive NAint M lineage at salt concentrations up to 20‰ (Mozdzer et al., 2010), it is primarily limited to oligohaline and mesohaline wetlands in the mid-Atlantic United States (Vasquez et al., 2005; Packett and Chambers, 2006; Mozdzer et al., 2010). However, in other regions of the United States, like New England and the Midwest, native North American populations are not limited by salinity and occur in brackish river systems as well as in saltmarshes (e.g., Burdick et al., 2001; Kettenring and Whigham, 2009; Kettenring and Mock, 2012; Cronin et al., 2015). These results indicate that salt tolerance is genotype-specific rather than lineage-specific, and highly variable within the species. Plant responses to changes in soil salinity can therefore be elucidated by studying locally adapted genotypes rather than lineages; the genetic composition of a population exposed to changing salinity regimes will be altered according to its pre-adaptation for salt tolerance (**Figure 2**).

Locally adapted genotypes may, however, not be the only strategy *P. australis* employs to persist in saline environments. Salt avoidance by below-ground labor division may also play a significant role in acclimation to shifts in salinity regime, as has also been found in the clonal species *Schoenoplectus americanus* in a brackish tidal wetland (Ikegami et al., 2008). An important component of *P. australis*’ ability to grow in soils spanning a wide range of salinities is its extensive rhizome and root system. Thus, most of the belowground biomass of lineages occurring in North America has been found in the upper 70 cm of soil (Moore et al., 2012), though depths can exceed 3 m even at coastal sites (Mozdzer et al., 2016b). This morphology may grant the species access to freshwater resources at soil depths less affected by tides. The importance of belowground organs to salt tolerance has also been demonstrated in Asia in landscapes with patchy soil salinity, where the genetic variation of *P. australis* is closely correlated with habitat heterogeneity (Gao et al., 2012).

Despite being able to survive and grow in saline soil conditions, *P. australis* has historically been considered a fresh to brackish water species (Raunkiaer, 1893; Haslam, 1973; Matoh et al., 1988). Several studies have identified negative effects of greater salinity levels on various traits including biomass production, culm height, stand density, culm diameter, and rhizome carbohydrate content (Lissner et al., 1999a; Engloner, 2009; Achenbach et al., 2013; Tang et al., 2013; Eller et al., 2014a). Physiologically, *P. australis* responses to high salinity are associated with decreases in tissue water potential, stomatal conductance and transpiration rates, photosynthetic efficiency of PSII, and nitrogen uptake rates (Chambers et al., 1998; Lissner et al., 1999b; Naumann et al., 2007; Pagter et al., 2009; Mozdzer et al., 2010; Zhang and Deng, 2012). The photosynthetic recovery and re-opening of stomata after short-term exposure to high salinity differs between genotypes of different lineages, demonstrating that sensing and responding to osmotic stress is a genotype-specific feature (Achenbach and Brix, 2014). Also, high-affinity K^+^ transporters isolated from salt tolerant reed plants are more efficient in K^+^ uptake and less permeable to Na^+^ than transporters from salt-sensitive plants, offering an explanation for their difference in salt-sensitivity (Takahashi et al., 2007).

Salinity increases in freshwater wetlands are likely to affect the natural distribution of *P. australis* genotypes and to alter the competitive dynamics between less and more salt-resistant plants. Spread of *P. australis* into salt marshes might also be accelerated in El Niño years due to temporary decreases in salinity from heavy rains that open windows for seedling establishment (Minchinton, 2002) and also due to the expansion of patches that maintain access to less saline groundwater in other parts of the stand. Storm surge from tropical storms, cyclones, and hurricanes can flood near-coastal freshwater wetlands and greatly elevate salinity levels. In North America, the spread of invasive NAint M populations is strongly positively correlated with the frequency of these storms and it has been argued that NAint M lineage thrives because it is more salt tolerant than native wetland plants (Burdick and Konisky, 2003; Bhattarai and Cronin, 2014). Unlike climatic adaptations that can be attributed, in part, to the phylogeographic origins of *P. australis* lineages, salt tolerance cannot simply be ascribed to a specific phylogenetic background, but is rather a consequence of the single pre-adapted genotype (Gao et al., 2012; Achenbach et al., 2013). Locally adapted genotypes of different reed lineages may therefore serve as models for studying responses to changes in soil salinity. Depending on the lineage, however, the outcome of interactions with other global change drivers can be estimated. Overall, future changes in *P. australis* salt tolerance are very likely, as responses to salinity have been shown to interact with temperature and CO_2_, and may confer greater salt resistance on *P. australis* due to improved osmotic acclimation and higher assimilation rates (Lissner et al., 1999a; Eller et al., 2014a). A lineage with inherently high phenotypic plasticity, such as NAint M, can be expected to benefit more from interactive effects of salinity and elevated CO_2_ than NAnat.

More research is needed to determine which genetic factors underlie the high salt tolerance of genotypes within *P. australis* lineages, and how and why these factors arise in these genotypes. Due to their increased likelihood of including salt-resistant genotypes, populations and stands with high genetic variability will probably have the strongest prospects of adapting to changes in salinity. Moreover, individual stands of *P. australis* will likely face genetic impoverishment following the extinction of salt-sensitive genotypes under shifting soil salinity regimes (**Figure 2**).

### Flooding Effects

Climate projections indicate that greater variability in precipitation will cause more frequent extremes in precipitation and discharge in many areas. This will increase the frequency and magnitude of inland and coastal floods, which will be compounded by larger storm surges and rising sea levels (IPCC, 2014).

Although *P. australis* seedlings are extremely vulnerable to flooding (Chambers et al., 2003; Mauchamp and Methy, 2004; Baldwin et al., 2010; Kettenring et al., 2015), once established, seedlings and adult plants are highly tolerant of inundation. Specifically, the survival, physiology, and growth of *P. australis* are less affected by submersion than are many other wetland plants (Gries et al., 1990; Brix et al., 1992; Armstrong et al., 1996b). Moreover, susceptibility to flooding decreases with ontogeny in the species (Bart and Hartman, 2003; Chambers et al., 2003; Whyte et al., 2008; Tulbure and Johnston, 2010). *P. australis* is also tolerant of greater amplitude fluctuations (±45 cm) in water level than other species, provided that its elevation is close to the mean water level (White et al., 2007). However, high water during extreme flooding years caused reed belts to decline along lakes in southern Germany and Austria; stands rejuvenated only in low-water years (Ostendorp, 1999; Ostendorp et al., 2003).

We found no studies that directly assessed genotypic differences in flooding tolerance. However, the number of genotypes represented in a lakeshore stand in Hungary decreased with water depth (Engloner and Major, 2011), which the authors attributed to genotype-specific flooding tolerance. Another study found that seasonal profiles of amino acids and carbohydrates differed by genotype and flooding regime in a German fen (Koppitz, 2004; Koppitz et al., 2004), though genotype by flooding regime interactions were not reported. These findings indicate that responses to flooding are a consequence of pre-adapted genotypes rather than adaptation at the lineage scale. Like soil salinity, plant responses to fluctuating water levels can best be studied in locally adapted genotypes, and the population response to be expected will be an alteration of its genetic composition (**Figure 2**).

Responses to flooding depend on the interaction of other drivers of global change in a way that is similar to soil salinity. For example, belowground, deeper water induces a shallower rhizome depth distribution (Weisner and Strand, 1996; Vretare et al., 2001; White and Ganf, 2002; Mozdzer et al., 2016b), but this rooting depth will deepen with rising CO_2_ concentrations (Mozdzer et al., 2016b). Vretare et al. (2001) suggested that *P. australis* increases allocation to stem height when growing in deeper water and simultaneously decreases stem density and belowground allocation. While stem density appears to be consistently lower in deeper water (Yamasaki and Tange, 1981; Vretare et al., 2001; Bodensteiner and Gabriel, 2003), stem height has been reported to both increase and decrease in response to deeper flooding (e.g., Hellings and Gallagher, 1992; Coops et al., 1996; Vretare et al., 2001; Engloner, 2004). Genotypic differences in the *P. australis* plants studied may contribute to the variation in outcomes from these studies, though this has not been assessed in the majority of cases. Facing more frequent flooding regimes with global change (IPCC, 2014), natural selection of flooding-resistant genotypes can be anticipated such that flooded populations may become genetically impoverished. Previous studies have often focused on the growth and morphological acclimation of *P. australis* to flooding, but there is a need to investigate the physiological consequences of inundation more thoroughly. Recent advances in research on photosynthesis in submerged shoots showed that elevated CO_2_ can alleviate flooding stress (Winkel et al., 2014). Although not investigated in *P. australis*, this capability would be especially relevant to determining seedling responses to inundation.

### Drought Effects

*Phragmites australis* is well-adapted for life in flooded environments but is tolerant of the full range of wetland hydrological conditions, including drought (Pagter et al., 2005). Wetland hydrology can be highly variable, with relatively dry conditions being common or even extreme in times of drought (Mitsch and Gosselink, 2007). With climate change, drought is predicted to develop more quickly and increase in intensity in many regions of the world (IPCC, 2014; Trenberth et al., 2014). *Phragmites australis* deals with drought through both short-term tolerance mechanisms (i.e., by making physiological or biochemical adjustments) and longer-term avoidance strategies that affect morphological and developmental traits (Morgan, 1984; Chaves et al., 2002; Pagter et al., 2005; Touchette et al., 2007). Following extreme low-water conditions, reed stands employ a “guerilla strategy” to efficiently and quickly occupy new wet habitats; they produce tillers across the uninhabited littoral zone as well as “legehalme,” which are rapidly elongating, horizontal shoots from which new culms emerge at the nodes (Ostendorp and Dienst, 2012).

Under dry soil conditions (*in situ*), *P. australis* substantially decreases leaf osmotic potential and accumulates more soluble sugars, amino acids, protein metabolites, proline, and nutrient elements than under moist conditions (Elhaak et al., 1993). When subjected to mild water stress, *P. australis* reduces total leaf area and biomass, but severe water stress induces changes in osmolality, leaf proline concentration, leaf chlorophyll *a* content, stomatal conductance, and photosynthetic rates (Pagter et al., 2005). Similarly, terrestrial dryland ecotypes of *P. australis* from northwest China increase their capacity for osmotic adjustment, significantly decrease stomatal conductance, reduce net photosynthetic rate, and their cover and height declines (Cui et al., 2010). Compared to wetland ecotypes, they also exhibit greater water use efficiency, increased activity of C_4_ photosynthetic enzymes, protective down-regulation of photosynthetic enzyme activities, and greater antioxidant enzyme activity (contributing to oxidative stress protection; Wang et al., 1998; Zhu et al., 2001, 2003a,b; Gong et al., 2011; Xiang et al., 2012). With the onset of complete drought (in controlled experimental studies), *P. australis* showed signs of drought in leaf xylem pressure potentials by the second day, stomatal conductance and photosynthesis by days four to eight, and leaf rolling and wilting by day five (Saltmarsh et al., 2006; Naumann et al., 2007). Field-based phenological studies of *P. australis* in Great Britain showed that years with spring drought can induce later emergence and flowering, as well as shorter culms, compared to years with normally flooding patterns. Also, years with fall drought may lead to earlier senescence compared to years when the stand is flooded. Nonetheless, *P. australis* rhizomes can penetrate up to 2 m into the soil to access deeper groundwater (Haslam, 1970).

As with salinity tolerance, it is very likely that some *P. australis* genotypes are more drought tolerant than others, even within lineages (**Figure 3**). However, drought events are more tightly coupled to climate than are high salinity periods, with the implication that drought-resistance could be phylogeographically determined in the species. It remains to be determined if phylogenetically determined drought tolerant lineages are also salt tolerant. For example, it seems that the North American native lineage is more sensitive to drought in some regions, such as the southwestern United States, where it is often associated with small streams and springs, which are sensitive to small changes in water availability (Meyerson et al., 2010a; Kettenring and Mock, 2012; Kettenring et al., 2012). In contrast, short-term drought that leads to temporary drawdowns may benefit colonization of the NAint M lineage by fostering seedling recruitment (Alvarez et al., 2005; Tulbure et al., 2007; Whyte et al., 2008; Kettenring et al., 2015, 2016). These studies serve as examples for using *P. australis* as model to study, whether physiologically similar responses to different global change factors result from similar adaptations or are independent of the plants’ phylogeographic origin.

### Eutrophication Effects

Increases in nutrients from atmospheric deposition, agriculture, and development are a well-known component of global change (Galloway et al., 2004). The ability of *P. australis* to efficiently take up nutrients, especially nitrogen (N; i.e., NO_3_^-^, NH_4_^+^, and dissolved organic nitrogen), suggests that increased eutrophication from human activities will have a positive impact on the spread of the species, particularly its invasive lineages. Wetland eutrophication is expected to increase in areas such as in agricultural and densely populated urban and suburban areas where nutrient loads continue to increase. However, the distribution of eutrophication under global change is likely to be spatially heterogeneous across regions. In some places, increased water resources due to glacier melting or increased precipitation may dilute N concentrations, whereas, in other places, evaporation and decreased precipitation could exacerbate the effects of pollutants and nutrients (IPCC, 2007, 2014).

Although *P. australis* grown under controlled experimental conditions generally responds positively to nutrient addition, e.g., displaying increased biomass production and a greater shoot density (Szczepanska and Szczepanski, 1976; Romero et al., 1999; Tho et al., 2016), eutrophication was a key factor responsible for reed die-back in Europe during the 1990s (van der Putten, 1997). However, the detrimental effects were predominantly indirect and caused by anoxic sediments, phytotoxin production from algal blooms or increased litter production, callus development and blockage of gas transport pathways in rhizomes and roots, and exacerbated by other human-induced impairments of natural reed habitats (Armstrong et al., 1996c; Brix, 1999b). In general, the high aeration capacity of the species allows for high root respiration rates throughout its large root systems, which, in turn, can facilitate high nutrient uptake rates (Nakamura et al., 2013). *Phragmites australis* lineages with inherently high biomass productivity and high belowground:aboveground ratios are therefore well-adapted for growth under increasingly eutrophic and anaerobic conditions, and appropriate models for investigating nutrient availability responses of highly productive and ruderal species (**Figure 3**). Increased nutrient availability is also likely to increase *P. australis* inflorescence and floret production (Kettenring et al., 2011) as well as seedling success given that seedlings will grow more rapidly beyond a vulnerable size (Saltonstall and Stevenson, 2007; Kettenring et al., 2015). Nutrient addition can also alter phenology, inducing culms to grow more rapidly early and late in the year, increasing their heights and annual carbon gains (Caplan et al., 2015). Relative to other wetland species, N affinity is very high for *P. australis*, but is usually its limiting nutrient (Chambers et al., 1998; Clevering, 1998; Romero et al., 1999; Saltonstall and Stevenson, 2007; Mozdzer et al., 2010). In contrast to phosphate, nitrate availability has been shown to result in altered aboveground:belowground biomass ratio of *P. australis* by favoring aboveground productivity with increasing N addition (Ulrich and Burton, 1985).

In North America, both native and introduced *P. australis* lineages have the capacity to rapidly take up and assimilate nutrients including inorganic N (Meyerson et al., 2000; Windham and Meyerson, 2003; Hazelton et al., 2010; Mozdzer and Zieman, 2010) and organic N (Mozdzer et al., 2010). However, most studies indicate that, in response to increased N availability, the NAint M lineage is competitively superior to many other wetland species (Chambers et al., 1998; Meyerson et al., 2000; Windham and Meyerson, 2003; Hazelton et al., 2010; Mozdzer et al., 2010, 2013) as well as to the native NAnat lineage (Saltonstall and Stevenson, 2007; Holdredge et al., 2010; Mozdzer et al., 2010, 2013; Mozdzer and Megonigal, 2012). This may be due to its ability to substantially increase carbon assimilation in response to greater N availability (Caplan et al., 2015). Nevertheless, NAint M is also able to regulate its N metabolism to outperform NAnat under low-N conditions (Mozdzer and Megonigal, 2012). This greater plasticity and ability to use available N in both eutrophic and oligotrophic ecosystems can enhance this lineage’s invasiveness by conferring traits such as shifts in phenology as well as increased height growth, leaf area, specific leaf area, leaf area ratio, root mass fraction, and foraging distance (Meadows, 2006; Holdredge et al., 2010; Mozdzer and Zieman, 2010; Mozdzer et al., 2010, 2013; Mozdzer and Megonigal, 2012).

In contrast to NAint M in North America, reeds from the East Asian/Australian group have lower plasticity, N uptake capacity and assimilation rates than co-occurring *Spartina alterniflora*, a C_4_ grass that displaces *P. australis* on the east coast of China (Zhao et al., 2010). Reed lineages adapted to nutrient-poor sites, which preferably translocate nutrients to storage organs rather than the assimilating tissue, may be outcompeted by stronger competitors for nutrients when in eutrophied settings. They may also respond by increasing productivity and culm height, which, due to their inherently lower tissue N allocation, may lead to poor culm stability and mechanical impairment (Kühl et al., 1997). Lineages that are capable of utilizing nutrients at higher concentrations, especially by allocating more biomass and N to their aboveground organs, may gain competitive advantages that contribute to invasive behavior in eutrophied habitats (Tho et al., 2016). The impacts of eutrophication on plants that are adapted to low vs. high nutrient availability can be studied by using NAnat and NAint M. Lineages EU and MED are also appropriate model systems to investigate eutrophication, although to a lesser extent than the North American lineages (**Figure 3**).

## Conclusion and Future Outlook

Although *P. australis* has been intensely studied, gaps in knowledge remain with respect to the effects that global change will have on community- and ecosystem-level processes. For example, it is not clear how global change will affect *Phragmites*-herbivore interactions under increased N availability, rising temperatures, and in some regions, increasing salinity (Cronin et al., 2015). Also, the increasing availability of phosphorus may ameliorate the susceptibility of *P. australis* to physiological stress induced by increased N availability (Tylová et al., 2013) and deserves further investigation. Additional research on wetland soil biogeochemistry and potential changes in nutrient availability under global change are also critically needed. For example, deep rooting by *P. australis* primes soil carbon deep within the soil profile, accelerating N mineralization under elevated CO_2_ and N conditions (Mozdzer et al., 2016b) and inducing a loss of previously recalcitrant soil carbon (Bernal et al., 2017). This may offset the concomitant stimulation to *P. australis*’ gross primary productivity (Caplan et al., 2015), such that the net effects on carbon storage potential of wetlands under global change are unclear. Moreover, there is a need to investigate the suggested modified photosynthetic pathway to compare responses to climate change of C_3_ and C_4_-like lineages, including gene-expression patterns and the role of photorespiration under elevated atmospheric CO_2_ (Bräutigam and Gowik, 2016). Warmer temperatures may increase the impact of *P. australis*-specific pathogens (Nechwatal et al., 2008), highlighting that climatic effects on pathogenic and symbiotic organisms in the rhizosphere, as well as their effects on the performance of *P. australis*, deserve further attention. Field investigations addressing higher trophic levels and changing soil conditions would extend future projections of the viability and range distribution of *P. australis* especially in the plant’s role as an ecosystem engineer affecting the role of wetland habitats as carbon sinks (Mitsch et al., 2013; Caplan et al., 2015).

As a species, *P. australis* has a high phenotypic plasticity, an extensive ecological amplitude, and capacity to acclimate to adverse environmental conditions. As such, *P. australis* is unlikely to be threatened by the multiple effects of global change in most regions, but can be expected to benefit from them in many cases. Here, the occurrence of strong latitudinal clines within and between *P. australis* lineages can be a useful tool for predicting climate change responses, specifically using populations within the same lineage that are distributed over a large geographical gradient. Adaptation to the climate of origin will confer these populations phenotypic plasticity to climatic drivers, and allow comparisons of climate change effects. Reverse transplant experiments and common gardens are particularly amenable to investigations of functional trait responses to climate change. This is demonstrated by reed lineages with distinct phylogeographic origins growing in similar environments, which respond differently to changes in climatic conditions. As global change will place intense selective pressure on diverse *P. australis* lineages, the distribution and interactions of co-occurring lineages and their within-population variability is very likely to be altered (**Figure 3**). The globally high genetic (Saltonstall, 2002; Lambertini et al., 2006, 2012a,b,c; Meyerson et al., 2010a, 2012), genomic (Suda et al., 2015; Meyerson et al., 2016b), and phenotypic diversity within *P. australis* suggests that both lineage- and genotype-specific responses to global change are likely to occur, resulting either in acclimation, advancement or range-shifts. We have distinguished four lineages that can be suitable models for plant species from higher latitudinal ranges (EU), lower-latitudinal ranges (MED), confined ecosystems (NAnat), and fast-spreading species with high phenotypic plasticity (NAint M) (**Figure 3**).

Although some stress tolerance mechanisms are genetically determined (e.g., those against flooding or salinity), they do not seem to be consistent within lineages. Hence, selection and differentiation within reed populations will be affected by their interactions with local environmental factors. In the worst case, a directional shift in the environment may result in genetic impoverishment of those populations or lineages with a few pre-adapted genotypes and few genotypes with inherently high phenotypic plasticity toward the specific global change driver (**Figure 2**). Ultimately, reduced genetic diversity may even lead to diminished population viability and local extirpation (Pauls et al., 2013). It is important to note that locally adapted populations that would otherwise be maladapted for rapidly changing future conditions may experience expanded gene-flow due to hybridization between lineages and could eventually replenish populations with genetic diversity.

The rapid invasion of non-native *P. australis* lineages across North America proves that a selection of well-adapted, highly plastic genotypes in a novel environment is possible and may occur elsewhere. The consequences for ecosystem functioning may be drastic and impossible to reverse. The replacement of diverse genotypes with a few well-adapted genotypes or lineages may yield strong competitors with traits promoting invasion; this may be difficult to detect and control in a species with a cosmopolitan distribution. As we have shown, the ecophysiological responses of *P. australis* to global change depend on the lineage and genotypes within it. We suggest that the phylogeographic background has to be considered when estimating the future distribution of *P. australis* populations and populations of cosmopolitan species in general.

## Author Contributions

All authors substantially contributed to the work. FE, DFW, and HB drafted the *Introduction*; CL drafted *Intraspecific Variation*; FE, JTC, and MKM drafted *Influences of Environmental Gradients*…; FE, BKS, TJM, and JSC drafted *Intraspecific Diversity Determines Responses*…; and FE, TJM, XG, W-YG, PP, HS, ELGH, JSC, BKS, KMK, LAM, JTC, MKB, and GPB drafted *Key Ecophysiological Processes*. All authors contributed to *Effects of Major Drivers*… and *Conclusion and Future Outlook*. FE, JSC, HS, CL, ELGH, HB, and BKS made final editorial adjustments. All authors commented on and edited the document and have approved the final submitted document.

## Conflict of Interest Statement

The authors declare that the research was conducted in the absence of any commercial or financial relationships that could be construed as a potential conflict of interest.
